# Small blood stem cells for enhancing early osseointegration formation on dental implants: a human phase I safety study

**DOI:** 10.1186/s13287-021-02461-z

**Published:** 2021-07-02

**Authors:** Sheng-Wei Feng, Yi-Han Su, Yen-Kuang Lin, Yu-Chih Wu, Yen-Hua Huang, Fu-Hung Yang, Hsi-Jen Chiang, Yun Yen, Peter Da-Yen Wang

**Affiliations:** 1 School of Dentistry, College of Oral Medicine, Taipei Medical University, Taipei, Taiwan; 2 Division of Prosthodontics, Department of Dentistry, Taipei Medical University Hospital, Taipei, 110 Taiwan; 3 Research Center of Biostatistics, Taipei Medical University, Taipei, 110 Taiwan; 4 School of Respiratory Therapy, College of Medicine, Taipei Medical University, Taipei, 110 Taiwan; 5 International PhD Program for Cell Therapy and Regeneration Medicine, College of Medicine, Taipei Medical University, Taipei, 110 Taiwan; 6 Research Center for Cell Therapy and Regeneration Medicine, Taipei Medical University, Taipei, 110 Taiwan; 7 Department of Biochemistry and Molecular Cell Biology, School of Medicine, College of Medicine, Taipei Medical University, Taipei, 110 Taiwan; 8 Research Center of Cancer Translational Medicine, Taipei Medical University, Taipei, 110 Taiwan

**Keywords:** SB cell therapy, Osseointegration, Dental implantation, Stem cells, Guided bone regeneration

## Abstract

**Background:**

Small blood stem cells (SB cells), isolated from human peripheral blood, demonstrated the ability to benefit bone regeneration and osseointegration. The primary goal of our study is to examine the safety and tolerability of SB cells in dental implantation for human patients with severe bone defects.

**Methods:**

Nine patients were enrolled and divided into three groups with SB cell treatment doses of 1 × 10^5^, 1 × 10^6^, and 1 × 10^7^ SB cells, and then evaluated by computed tomography (CT) scans to assess bone mineral density (BMD) by Hounsfield units (HU) scoring. Testing was conducted before treatment and on weeks 4, 6, 8, and 12 post dental implantation. Blood and comprehensive chemistry panel testing were also performed.

**Results:**

No severe adverse effects were observed for up to 6-month trial. Grade 1 leukocytosis, anemia, and elevated liver function were observed, but related with the patient’s condition or the implant treatment itself and not the transplantation of SB cells. The levels of cytokines and chemokines were detected by a multiplex immunological assay. Elevated levels of eotaxin, FGF2, MCP-1, MDC, and IL17a were found among patients who received SB cell treatment. This observation suggested SB cells triggered cytokines and chemokines for local tissue repair. To ensure the efficacy of SB cells in dental implantation, the BMD and maximum stresses via stress analysis model were measured through CT scanning. All patients who suffered from severe bone defect showed improvement from D3 level to D1 or D2 level. The HU score acceleration can be observed by week 2 after guided bone regeneration (GBR) and prior to dental implantation.

**Conclusions:**

This phase I study shows that treatment of SB cells for dental implantation is well tolerated with no major adverse effects. The use of SB cells for accelerating the osseointegration in high-risk dental implant patients warrants further phase II studies.

**Trial registration:**

Taiwan Clinical Trial Registry (SB-GBR001) and clinical trial registry of the United States (NCT04451486).

**Supplementary Information:**

The online version contains supplementary material available at 10.1186/s13287-021-02461-z.

## Background

The current treatments for a missing tooth or teeth include dental implants, dental bridges and dentures [1]. Even though dental implant procedures have high clinical success rates and are widely used, their success depends highly on the condition of the surrounding bone [2, 3]. If the healing ability of this surrounding bone is compromised after tooth extraction, the success rate of restoration is diminished [4]. Guided bone regeneration (GBR) is one technique that is commonly to enhance osseointegration of the surrounding alveolar bone after extraction [5, 6]. During GBR, a barrier membrane is applied to exclude non-osteogenic tissue and promote osteogenesis and osteoconduction [7]. The reconstructive procedure is done often in combination with bone grafting material such as mineralized bone allografts. Stem cells have also been proposed as another way to enhance GBR [8–11].

A novel type of stem cells called SB cells have been previously reported [12]. These stem cells can be isolated from human adult blood, bone marrow, or fetal cord blood. SB cells share some characteristics to embryonic stem cells with superior plasticity and the ability differentiate into cell types of the mesoderm including, but not limited to osteocytes, chondrocytes, and adipocytes, as well as neurons (ectoderm), liver cells (endoderm), and muscle cells (including cardiomyocytes). Several types of other stem cells that are similar characteristics to SB cells include VSELs (very small embryonic-like stem cell; CD133+) [13], MSCs (mesenchymal stem cell; Stro-1+) [14], and BLSCs (blastomere-like stem cell; CEA+) [15]. The SB cells derived from human adult blood are called small blood stem cells. These cells have additional advantages in regenerative medicine including easy access for harvesting, distinguishable surface markers, and the ability for self-renewal [12]. As described in an early protocol, more than 1 × 10^7^ SB cells were isolated and purified from 20 mL of patient blood [12]. Distinct features of these stem cells include (1) the cell sizes in suspension were between 0.3 and 6.0 μm, preferably 0.5 to 5.0 μm, (2) the presence of cell surface markers such as Lgr5+, CD61-, and Lin- and (3) DAPI- or SYTO-positive staining (to exclude platelets and extracellular vesicles, which lacked nuclei). Preclinical animal studies have also demonstrated that SB cells do not form teratomas in vivo, suggesting their low risk of tumor formation (unpublished results, manuscript in preparation). As part of an autologous therapy, SB cells would also not be expected to cause immune-mediated rejection when infused into patients.

Additionally, SB cells have been tested in various animal models for their ability to heal bone and diabetic wound healing. The potential benefits of SB cells in bone regeneration and osseointegration were observed in mouse and rabbit models (manuscript in preparation) [16]. A case study of a patient with a dental implant coated with SB cells that were derived from bone marrow has also reported [17]. Here, we present a human phase I study to examine the safety and tolerability of using autologous small blood SB cells to ameliorate GBR in dental implant patients.

## Materials and methods

### Clinical trial design

This study was registered in the Taiwan Clinical Trial Registry (https://www.cde.org.tw; SB-GBR001) and clinical trial registry of the USA (https://clinicaltrials.gov/; NCT04451486). The protocol was reviewed and approved by a joint Institutional Review Board (IRB) at Taipei Medical University Hospital (TMUH; N201709009) and the Taiwan Food and Drug Administration (TFDA; 1060037830), and conducted in accordance with Good Clinical Practice standards under the regulation of TFDA. No deviation from the protocol was implemented without prior review and approval of the IRB except in cases where it was necessary to eliminate an immediate hazard to a research subject. In such case, the deviation would be reported to the IRB as soon as possible. All patients were provided a consent form describing the study and given sufficient information to make an informed decision about their participation.

For determination of sample size, sequential cohorts in a “3 + 3 design” dose escalation trial were applied in our phase I clinical trial (first-in-human). In brief, in a “3 + 3 design,” three patients are initially enrolled into a low dose. If there is no dose-limiting toxicity (DLT) observed in any of these patients, the trial proceeds to enroll three patients into the next higher dose. If one patient develops a DLT, additional three patients are needed in the same dose. This trial design is based on FDA guidance and previous studies, which would expose a minimal number of participants to potential doses, maintain safety and determine the maximum tolerated dose [18–23]. Therefore, a minimum of nine patients, in total, was needed to establish the safety profile of this phase I study. The first three patients were assigned to receive a low dose of SB cells (1 × 10^5^ CD61^−^Lin^−^ cells /0.25 mL DPBS), the three patients receive middle dose of SB cells (1 × 10^6^ CD61^−^Lin^−^ cells /0.25 mL DPBS), and the three patients receive high dose of SB cells (1 × 10^7^ CD61^−^Lin^−^ cells /0.25 mL DPBS).

Dental procedures were performed at TMUH. Patients were evaluated by accredited dentists and underwent a complete prosthodontic diagnostic evaluation by cone beam CT scan (GE Light Speed, GE® Healthcare) [24]. All patients who were eligible for this study suffered from severe bone defects as indicated by high D2-D3 levels using the Hounsfield units (HU) scale [2]. Changes in bone mineral density (BMD) were measured from week 0 as a baseline to week 24 [25]. Hard tissue evaluation was done on weeks 1, 2, 8, 12, 16, 18, 20, and 24 after GBR. Any detection of dental periapical changes from baseline have been measured. Detected changes from the baseline in the osseointegration region of interest (ROI) parameters have been recorded [1, 2, 4].

### Inclusion criteria

Patient eligibility was based on the following criteria: (1) Subject’s age is ≥ 20 years old; (2) Informed consent form is signed; (3) Subject has ≥ 1 missing tooth with D2, D3 bone density; (4) Subject has a gums environment defined by an alveolar bone height of ≥ 10 mm and a bone width of ≥ 8 mm; (5) Subject has one missing maxillary or mandibular posterior tooth that requires a GBR prior to a dental implant; (6) Bone defect(s) is present with at least two walls missing; (7) The opposing dentition are natural teeth, fixed crowns on natural teeth or bridges on natural teeth, or implants. Removable prostheses or dentures opposing the study implants are not allowed; (8) Subject is able to understand and comply with requirements, instructions, and restrictions stated in the protocol. In addition, the patient’s periodontal health status is evaluated as follows: healthy periodontal conditions of the neighboring teeth; good oral hygiene defined as a full-mouth bleeding score lower than 25%, and a full-mouth plaque score ≤ 25%

### Safety evaluation

Safety was evaluated on the following criteria: (1) adverse events (AEs) up to 24 weeks after GBR; (2) vital signs recorded on weeks 0, 8, 12, and 24 after GBR; (3) laboratory tests including comprehensive biochemistry laboratory tests, complete blood count, and urine analysis conducted on weeks 8, 12, 16, and 24 after GBR; (4) immunological tests conducted on weeks 8, 12, 16, and 24 after GBR. AEs were graded using the NCI Common Terminology Criteria for Adverse Events version 3.0.

### Purifications of CD61^−^Lin^−^ SB cells

SB cells for autologous transplantation were isolated and purified from patient blood samples according to a modified protocol that was previously described [12]. In this trial, around 40 mL of subject’s blood was collected into four EDTA-containing anti-clotting tubes and stored at 4 °C. After 48–72 h, the blood samples were separated into two layers in a closed system. The top layer, which contained the SB mixture, was pipetted into a 50-mL tube, then centrifuged at 300×*g* for 15 min. After removing the supernatant, the pellet was washed with 10 mL DPBS, and transferred into 15-mL tubes, and then centrifuged at 300×*g* for 15 min. Mature hematopoietic cells were depleted from the SB mixture using a human lineage depletion kit (Miltenyi Biotec) and CD61^+^ cells were depleted using microbeads conjugated with anti-CD61 antibody (Miltenyi Biotec). The CD61^−^Lin^−^ SB cells were analyzed by flow cytometry to confirm the cell size and Lgr5^+^ population.

The CD61^−^Lin^−^ SB cell product was stored at 4 °C. Excursions were permitted between 2 and 8 °C. Each cell solution prepared for this trial was used within 24 h from completion of the final product. All procedures followed Good Tissue Practice (GTP) standards under the regulations of the TFDA.

### Quality control of the CD61^−^Lin^−^ SB cells product

The quality control assessment of the CD61^−^Lin^−^ SB cells product included cell size identification, cell viability, Lgr5^+^ SB cells counting, and safety assessment under TFDA-accredited GTP laboratory guidelines. Any CD61^−^Lin^−^ SB cell product that did not pass all the release criteria was destroyed. The CD61^−^Lin^−^ SB cell product was assessed on the following criteria: (1) negative mycoplasma culture test, (2) negative bacterial and fungal sterility test, (3) endotoxin ≤ 5 EU/ml, (4) cell viability of > 80%, (5) ≥ 2.5% Lgr5^+^ cells, (6) ≥ 95% of cells have a diameter between 2 and 5 μm, (7) appropriate concentrations of the CD61^−^Lin^−^ SB cell product was diluted in 0.25 mL of DPBS.

### Treatment administration CD61^−^Lin^−^ SB cells

The surgical site was cleaned according to standard dental procedure. The preparation of surgical sites was standardized across sites in all subjects according to local dental practice or as stated in the provisions of the protocol. The SB cells and hydroxyapatite powder (APACERAM Bone Graft Substitute, medical device 024045) were added to the wound area and covered with an absorbable double layer collagen membrane (Geistlich Bio-Gide®, medical device 021178). Three patients were enrolled per treatment dose: Low Dose Group 1 patients received 1 × 10^5^ CD61^−^Lin^−^ cells /0.25 mL DPBS; Middle Dose Group 2 patients received 1 × 10^6^ CD61^−^Lin^−^ cells /0.25 mL DPBS, and High Dose Group 3 patients received 1 × 10^7^ CD61^−^Lin^−^ cells/0.25 mL DPBS. The surgical sutures were applied to the wound and cleaned as standard procedure.

### Combination therapy of GBR collagen complex with CD61^−^Lin^−^ SB cells

As shown in Fig. S1, a full-thickness flap preparation was executed on the surgical site under local anesthesia (4% Ubistesin or Mepivastesin) [26]. The GBR collagen complex was placed in position according to the recommendations of the manufacturer [5]. After removing the granulation tissue, the alveolar bone defects were grafting with the SB cells and hydroxyapatite powder (APACERAM Bone Graft Substitute, medical device 024045) (Fig. S1a, b). After that, the wound area was covered with an absorbable double layer collagen membrane (Geistlich Bio-Gide®, medical device 021178) and stabilized with a suture. Twelve weeks after GBR surgery, implant sites were prepared and Osseotite® double acid-etched implants (Biomet 3i, Palm Beach Gardens, FL, USA) were placed in accordance with the manufacturer’s guidelines (Fig. S1d). Healing abutment was screwed onto the implant and left the healing abutment exposed to the oral environment [26, 27] (Fig. S1e). After 8 weeks of healing, definitive metal-ceramic crown was delivered (Fig. S1f). The whole assessment schedule in this clinical trial is presented in Fig. S2.

### Cytokine and chemokine testing

The peripheral blood was drawn at designated time points with patient consent. All samples were batched and kept at 4 °C before testing under one licensed medical technologist. The 48 Cytokines Multiplex Immunoassay Panel (Bio-Rad Laboratory Inc.) was used according to the manufacturer’s protocol. Statistical analysis was done by Dr. Yen-Kung Lin at TMU.

### Statistical analysis

Descriptive analysis on changes and comparisons between groups from baseline to endpoint were performed for soft and hard tissue evaluation: CT, BMD/RFA/BVF/stability; abutment and crown stability/torque, blood examination: cytokine/chemokines. Continuous variables are presented as mean, median (range). Categorical variables are displayed as counts. The slope as average change for cytokines and chemokines was examined with two-tailed t test. The overall responses for the three dosage groups were compared using one-way ANOVA with Tukey’s post hoc analysis. Significance was set at 0.05. Statistical software SAS 9.4 and R 2.15 were used for analysis.

## Results

### Purification and characterization of CD61^−^Lin^−^ SB cells

**Fig. 1:**
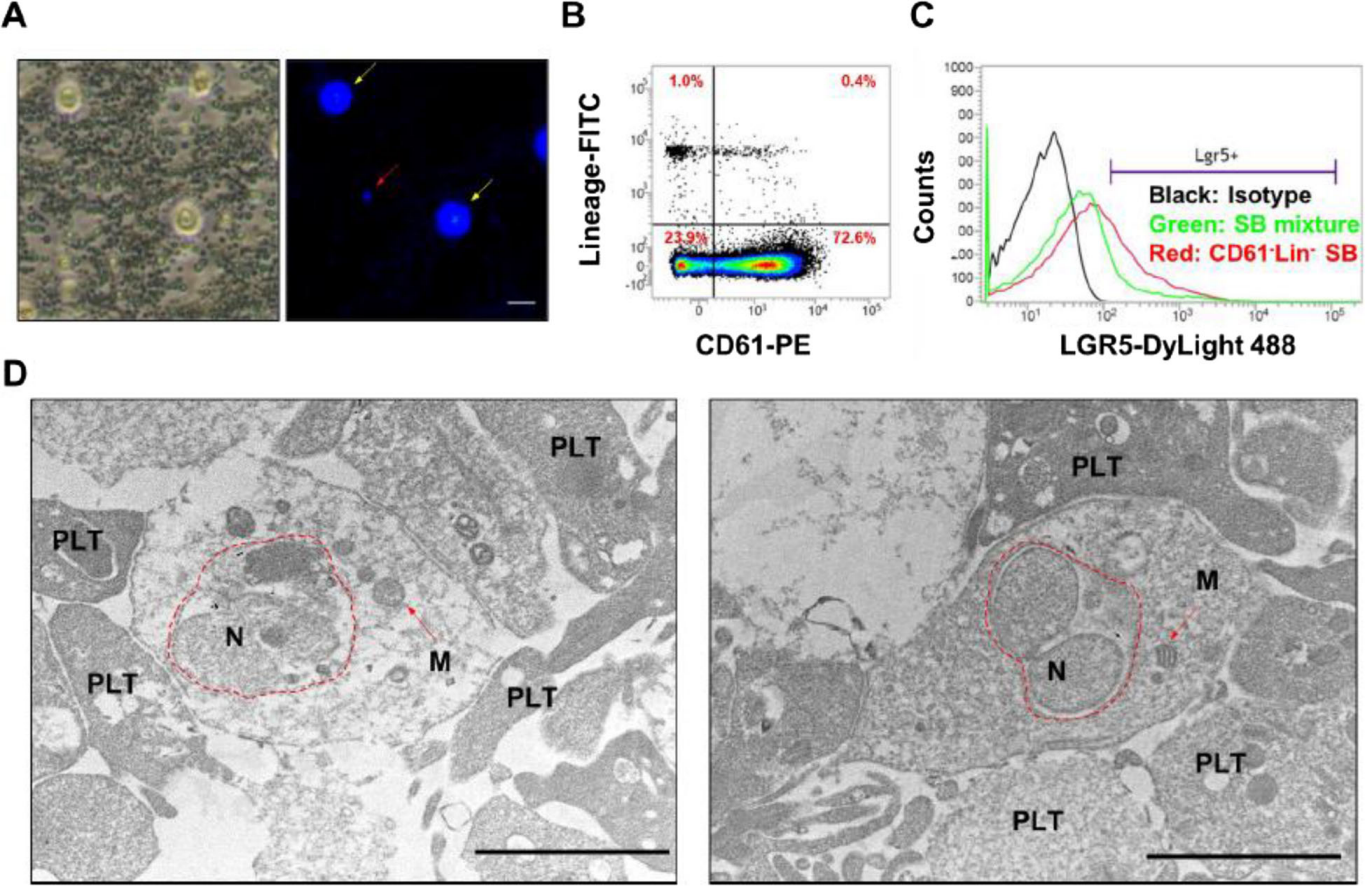
Purification and characterization of SB cells from human peripheral blood. **A** SB cells were presented using the DAPI positive staining (red arrow). WBC, yellow arrow. Bar = 10 μm. **B** The ratio of CD61^−^Lin^−^ cells in the SB mixture was analyzed by a flow cytometer. **C** The ratio of Lgr5^+^ cells in SB mixture or CD61^−^Lin^−^ fraction was analyzed by a flow cytometer. **D** SB cell morphology was analyzed by TEM. Red dotted line, nucleus; Red arrow, mitochondria. Bar = 2 μm

Our results indicated that a few population of the SB mixture cells were DAPI^+^ cells. It means the small (smaller than 6 μm in diameter) DAPI^+^ cell population could exclude platelets and extracellular vesicles, which lacked nuclei (Fig. 1a). In order to enrich the small DAPI^+^ cells, the platelet marker CD61 and mature hematopoietic lineage markers (Lin) were used to deplete the platelets and mature hematopoietic cells from the SB mixture layer. As shown in Fig. 1b, the SB mixture layer contains 23.9% CD61 and Lin double negative population. After using CD61 and Lin magnetic negative selection form SB mixture, the population of Lgr5^+^ cells in CD61^−^Lin^−^ fraction (as call CD61^−^Lin^−^ SB cells) increased 5.6 ± 2.3 fold compared to the population of Lgr5^+^ cells in SB mixture (Fig. 1c). In order to confirm the SB cell structure, the CD61^−^Lin^−^ SB cells were analyzed by using TEM. As shown in Fig. 1d, the cells have shown small cell size and the complete structure of nucleus and mitochondrion. These results demonstrate that CD61 and Lin magnetic negative selection method can enrich the population of Lgr5^+^ cells, and then the CD61^−^Lin^−^ SB cells were used in the following experiments. Furthermore, in our mouse and rabbit animal experiments, SB cells revealed low risk of tumor formation and non-tumorigenicity and promote new bone formation (Fig. S3, S4, S5).

### Clinical characteristics

A total of nine patients were enrolled. Three received a low dose of 1 × 10^5^ cells, three received a middle dose of 1 × 10^6^ cells, and three received a high dose of 1 × 10^7^ cells. The median age of patients was 54 years old with a range of 29 to 81 years old. The male to female ratio was 5:4. Patients presented with no major comorbidities. Some were hypertensive, but with good control. All presented with severe defects as indicated by D3 bone density levels. The background of patients and their conditions of dental defects have been summarized in Table 1.

**Table 1:** Clinical characteristics

Case no.	Age	Gender	Dose	Missing tooth	Category
			(Conc. of CD61-Lin- SB cells /0.25 mL DPBS)	Position no.	
1	60–69	2	Low (1 × 10^5^)	15	D3
2	70–79	1	Low (1 × 10^5^)	14	D3
3	40–49	1	Low (1 × 10^5^)	47	D3
4	20–29	1	Medium (1 × 10^6^)	35	D3
5	50–59	2	Medium (1 × 10^6^)	46	D3
6	50–59	2	Medium (1 × 10^6^)	26	D3
7	80–89	2	High (1 × 10^7^)	37	D3
8	40–49	1	High (1 × 10^7^)	36	D3
9	40–49	2	High (1 × 10^7^)	36	D3

Abbreviations: *Conc.* concentration, *DPBS* Dulbecco’s phosphate buffer solution, *No.* number

**Fig. 2:**
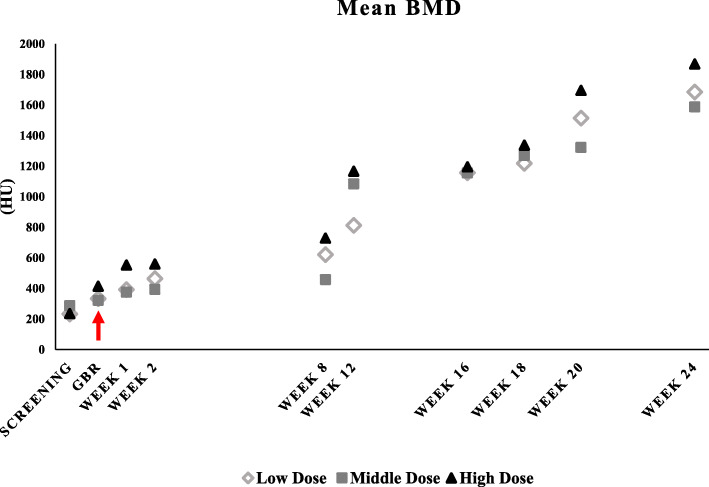
Mean bone mineral density (BMD) measurements. Patients were screened and stratified into three treatment dose groups: low (n = 3), middle (n = 3), and high (n = 3). The course of study spanned 24 weeks. Data points represent BMD measurements given as Hounsfield units (HU). “↓” indicates start of treatment and GBR. Dental implantation was done on week 12. The increase in BMD between dose groups were not statistically significant

The bone mineral density (BMD) was measured by computer tomography (CT) scans during the enrollment period and immediately prior to GBR procedure on week 0. CT imaging was also done during follow-up visits, including before dental implantation on week 12. Local anesthesia was given to all patients for surgical incisions and transplantation of SB cells covered with the collagen membrane. All procedures followed standard practice with no deviations in the study protocol. For a baseline, individual patient HU scores were recorded up to 12 weeks prior to surgical evaluation, and up to 12 weeks after dental implantation as indicated in Table 2. Bone quality was monitored and evaluated using CT attenuation coefficients with HU scores. The mean BMD for each of the three dose groups is presented in Fig. 2, and the BMD of individual patients is listed in detail in Table 2. A positive trend can be observed for all three groups. All dental implantations were considered successful.

**Table 2:** Bone mineral density of individual patients

Visit no.	Visit type	Case 1	Case 2	Case 3	Case 4	Case 5	Case 6	Case 7	Case 8	Case 9
		(HU)	(HU)	(HU)	(HU)	(HU)	(HU)	(HU)	(HU)	(HU)
1	Screening	330. 0	170.0	198.0	207.0	405.0	252.0	201.0	129.0	376.0
3	Guided bone regeneration (GBR)	330. 0	443.0	224.9	247.0	475.0	243.0	280.0	460.0	504.0
4	1 week follow-up after GBR	328.0	571.0	277.0	251.0	481.0	393.0	550.0	697.0	412.0
5	2 week follow-up after GBR	400.0	636.0	354.0	309.0	481.0	391.0	616.0	597.0	465.0
7	8 week follow-up after GBR	411.0	892.0	561.0	422.0	489.0	460.0	555.0	717.0	916.0
8	12 week follow-up after GBR—implantation	598.0	1094.0	745.0	550.0	1158.0	1542.0	839.0	907.0	1755.0
10	4 week follow-up after implantation	756.0	1773.0	942.0	915.0	1547.0	1004.0	939.0	717.0	1929.0
11	6 week follow-up after implantation	833.0	1887.0	935.0	1184.0	1609.0	1017.0	954.0	999.0	2058.0
12	8 week follow-up after implantation—abutment and crown	949.0	2410.0	1183.0	1239.0	1746.0	983.0	1224.0	1853.0	2012.0
13	12 week follow-up after implantation	1057.0	2622.0	1375.0	1893.0	1820.0	1050.0	1124.0	1982.0	2499.0

Abbreviations: *HU* Hounsfield units, *GBR* general bone reconstruction

### Safety and treatment-related adverse events

The overall occurrence of adverse events in patients treated with SB cells has been summarized in Table 3. All patients were evaluated for toxicity after transplantation of SB cells. Treatment was well tolerated with no serious adverse events (AEs). All were grade 1. AEs were distributed among a few patients. Patient 9 had a slightly elevated level of liver alanine aminotransferase (ALT) at the beginning of the study. There was no worsening during the total assessment period of six months. Patient 2 started with hemoglobin (Hb) level at 11.8 g/dL which recovered to normal levels (normal range 12–16 g/dL) after SB treatment on week 12. Patient 8 had an Hb level of 13 g/dL that decreased to 11.7 g/dL on month 4 and returned to 13 g/dL on the last recorded follow-up. Patient 6 presented normal white blood cells which were slightly elevated to 11.49 × 10^3^ cells/μL (compared to the normal range of 4.00–11.00 × 10^3^ cells/μL). Both patient 7 and patient 8 started with borderline high sugar levels at about 107 mg/dl (compared to the normal range of 70–99 mg/dl) that was slightly elevated to 117 mg/dl, but returned to baseline in a few weeks. There were no other adverse effects observed in hematological tests, the comprehensive chemistry panel, and urine analysis. Limited Grade 1 adverse events were therefore determined as not related to SB administration and treatment.

**Table 3:** Individual adverse events from total patient population (n = 9)

	Grade 1	Grade 2	Grade 3	Grade 4	Tx-related
Hematology					
Platelets	0	0	0	0	0
Hemoglobin	1	0	0	0	0
Abnormal leukocyte	1	0	0	0	0
Abnormal lymphopenia	0	0	0	0	0
Abnormal neutrophils	0	0	0	0	0
Non-hematology					
Bilirubin	0	0	0	0	0
AST	0	0	0	0	0
ALT	1	0	0	0	0
Sugar (AC)	2	0	0	0	0

Abbreviations: *AST* aspartate aminotransferase, *ALT* alanine aminotransferase, *Tx* treatment

### Profiling of cytokines and chemokines

Stem cells are known to trigger different cytokines and chemokines as a mechanism of action. To study the cytokines and chemokines released after SB cells administration, blood was collected and analyzed to examine the individual and group difference among all three doses. Results are summarized in Table 4.

**Table 4:** Cytokines and chemokines changes

Low dose			Middle dose			High dose		
var	avg_change	p_value	var	avg_change	p_value	var	avg_change	p_value
EGF	12.94	0.082	EGF	− 1.33	0.6061	EGF	16	0.1777
FGF2	− 0.83	0.7778	FGF2	5.18	0.0035*	FGF2	3.14	0.0581
Eotaxin	4.92	0.1053	Eotaxin	2.76	0.0043*	Eotaxin	4.72	0.3712
TGF_alph	0.14	0.4226	TGF_alph	0.21	0.4226	TGF_alph	0	1
G_CSF	0.64	0.4226	G_CSF	10.48	0.288	G_CSF	0.6	0.7915
Flt_3L	0	1	Flt_3L	0	1	Flt_3L	1.38	0.4226
GM_CSF	− 0.5	0.4226	GM_CSF	1.01	0.1904	GM_CSF	0.01	0.99
Fractalk	− 19.1	0.0104*	Fractalk	− 0.44	0.4226	Fractalk	8.77	0.4226
IFN_alph	0	1	IFN_alph	0.22	0.4226	IFN_alph	0.26	0.4226
IFN_gamm	− 0.85	0.3704	IFN_gamm	− 1.32	0.4114	IFN_gamm	1.49	0.4382
GRO	77.88	0.3749	GRO	47.98	0.2373	GRO	− 2.21	0.9386
IL_10	0	1	IL_10	− 1.45	0 4802	IL_10	0	1
MCP_3	0	1	MCP_3	3.66	0.2609	MCP_3	0.86	0.4226
IL_12p40	0	1	IL_12p40	0	1	IL_12p40	0	1
MDC	22.33	0.3761	MDC	55.89	0.0446*	MDC	5.01	0.8816
IL_12p7 0	0	1	IL_12p7 0	0.53	0.4226	IL_12p7 0	0.26	0.4226
IL_13	0	1	IL_13	− 0.37	0.8552	IL_13	1.06	0.4624
IL_15	0	1	IL_15	0.29	0.4226	IL_15	0.06	0.4226
sCD40L	1714.04	0.2155	sCD40L	488.89	0.2467	sCD40L	594.57	0.0859
IL_17A	− 0.83	0.2321	IL_17A	− 0.56	0.1837	IL_17A	0.17	0.8197
IL_1RA	− 1.93	0.672	IL_1RA	− 0.57	0.8492	IL_1RA	0.8	0.4226
IL_1alph	0	1	IL_1alph	0.68	0.9572	IL_1alph	7.64	0.4226
IL_9	0	1	IL_9	− 0.09	0.4226	IL_9	0.34	0.4226
IL_lbeta	0	1	IL_lbeta	0	1	IL_lbeta	0	1
IL_2	0	1	IL_2	− 0.22	0.4226	IL_2	0	1
IL_3	0	1	IL_3	0	1	IL_3	0	1
IL_4	6.48	0.315	IL_4	23.67	0.4287	IL_4	11.83	0.4226
IL_5	− 0.35	0.4226	IL_5	− 0.34	0.4226	IL_5	0.26	0.4226
IL_6	0	1	IL_6	2.2	0.3444	IL_6	1.16	0.4226
IL_7	− 0.48	0.3651	IL_7	1.54	0.1542	IL_7	− 0.86	0.5098
IL_8	0.7	0.2546	IL_8	1.8	0.0705	IL_8	0.77	0.556
IP_10	13.45	0.4772	IP_10	− 0.6	0.9709	IP_10	− 16.72	0.2174
MCP_1	21.64	0.0008*	MCP_1	15.45	0.0039	MCP_1	12.5	0.5057
MIP_lalp	0.85	0.4452	MIP_lalp	− 1.36	0.1323	MIP_lalp	3.11	0.3513
MIP_lbet	3.73	0.3903	MIP_lbet	9.02	0.0989	MIP_lbet	5.28	0.2209
TNF_alph	1.22	0.2699	TNF_alph	− 0.74	0.0006	TNF_alph	4.87	0 4082
TNF_beta	0	1	TNF_beta	4.28	0.3584	TNF_beta	1.52	0.4226
VEGF	− 3.67	0.651	VEGF	20.11	0.1275	VEGF	7.56	0.2767

Abbreviatlons: *var* variable, *avg* average

* indicates *p* < 0.05

**Fig. 3:**
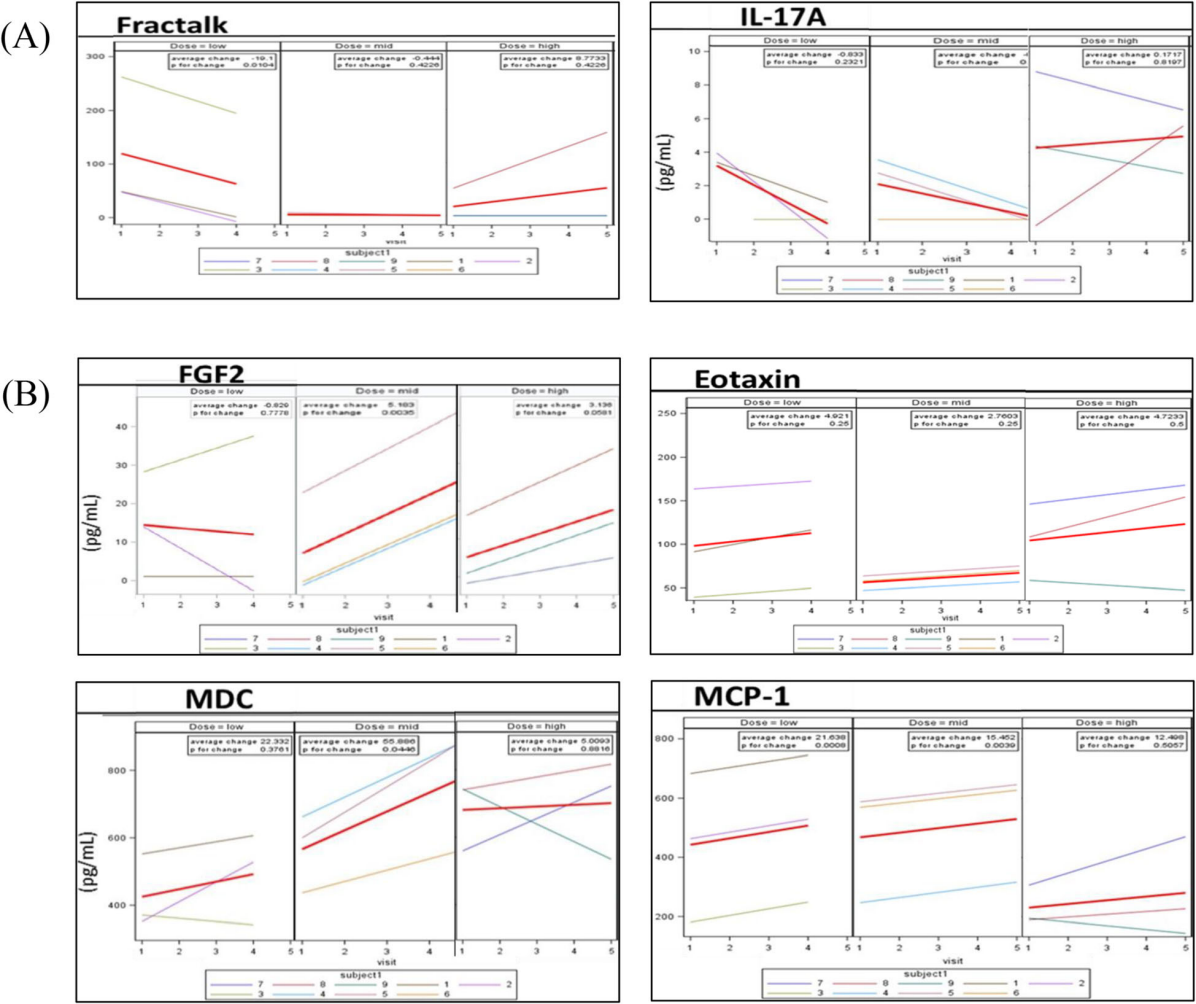
Temporal profiling of selected cytokines for individual patients based on dose groups (low, middle, and high). Blood was drawn from patients on week 4, immediately prior to the dental implant procedure on week 12 and during follow-up visits on weeks 16 and 24. Chemokine level was regressed over time using ordinary linear regression to estimate changes over time. Regression testing was done to estimate the profiles of Fractalkine (Fracktalk), interleukin-17A (IL-17A), fibroblast growth factor 2 (FGF2), eotaxin, macrophage-derived chemokine (MDC), and monocyte chemoattractant protein-1 (MCP-1). Results are given as concentration levels (pg/mL) on y-axis over time x-axis. Bolded red line represents β from generalized estimating equation

To evaluate the individual trajectories of cytokines and chemokines, each response was regressed on time using ordinary linear regression. The estimated change for all visits was calculated from the regression slopes. The linear cytokine expression trajectory for each patient was plotted and the selected six cytokine/chemokine profiles [Fractalkine (Fracktalk), interleukin-17A (IL-17A), fibroblast growth factor 2 (FGF2), eotaxin, macrophage-derived chemokine (MDC), and monocyte chemoattractant protein 1(MCP-1)] are presented in Fig. 3. Fracktalk and IL-17A showed decreasing tendency in all groups (Fig. 3a), while FGF2, eotaxin, MDC, and MCP-1 exhibited increasing tendency in all groups (Fig. 3b). Longitudinal variation for each visit was given both on an individual level and dose group level.

To further evaluate cytokine expression and consider confounding factors due to dosage, cytokine expressions regressed on timing of visit were stratified by treatment dosage. The regression coefficient β was considered as the average change or increment over each time points. As shown in Table 4, most cytokines and chemokines showed non-significant alternations in plasma levels during the 24-week follow-up. Among the cytokines and chemokines, a total of five biomarkers (Fracktalk, FGF2, eotaxin, MDC, and MCP-1) showed significant alternations. For the low dose group, the expression for MCP-1 increased by an average of 21.64 pg/mL for every consecutive visit (t = 35.685, p = 0.001). The expression for Fracktalk decreased by an average of 19.1 pg/mL at every consecutive visit (t = − 9.708, p = 0.01). For the middle dose group, FGF2 significantly increased by an average of 5.18 pg/mL (t = 16.806, p = 0.004). Significant increases were also observed for eotaxin, MDC, and MCP-1. For the high dose group, FGF2 and CD40L seemed to have an upward trend; however, the magnitude was not statistically significant. Treatment dose effect on cytokine expression was also examined before treatment and at the end of the study. Overall, there was no difference among the three groups. At the end of the study, IL-17A appeared slightly lower in patients given a middle or low dose of SB cells (F = 11.84, p = 0.008).

## Discussion

Bone quantity and quality are critical factors for the success of dental implants. The osseointegration between the implant and the surrounding bone plays a crucial role in implant stability. In this report, we study the application of SB cells as an autologous stem cell treatment prior to performing dental implantation. There were no adverse events observed related to the autologous transplantation of up to 1 × 10^7^ SB cells. Observations were based on hematologic data and non-hematologic data that was collected throughout the total period of 6 months.

All patients were considered to have favorable outcomes in this study. The successful osseointegration of dental implants is influenced by many factors [1, 2, 4]. Bone regeneration and stability at the implant-tissue interface depend on trabecular bone density as well as cortical bone density. Therefore, measurements of increasing BMD and maximum values of stress would partially represent the bone quality and quantity changes of treatment [4]. In this study, the posterior mandible known as the higher mean trabecular non-cortical bone density was evaluated for a D2–D3 level of bone density. The selection of only D3 level patients is remarkable because higher density levels correlated with lower success rates in dental implantations. More extensive analysis such as on the cortical bone thickness via biopsy or the microarchitecture of trabecular bone would provide better insight into the observed osseointegration in this study [2, 26, 27].

The results in this study suggest that SB cells can be applied safely to human patients with severe alveolar bone defects. The safety and tolerability presented here may also apply to other oral surgeries that require facilitation of bone remineralization in a timely manner. Current dental practice sometimes uses recombinant human bone mineralization protein-2 (BMP-2) for bone regeneration in patients [28, 29]. BMP-2 belongs to the TGF-beta superfamily and can promote bone and cartilage remodeling and growth. As a strong bone inductive cytokine, however, BMP-2 carries side effects such as ectopic bone growth, pain, and swollenness in nasal treatment and is costly to manufacture [29]. Other BMP peptides, such as BMP-4 and BMP-7, have also been studied for the same indication, but share similar drawbacks as BMP-2 when used in a clinical setting. Therefore, autologous SB cell therapy offers an alternative. As described in this study, the tolerability of SB cells would provide less pain and more comfort by reducing inflammation and could possibly replace BMP-2 in dental implant practice.

**Fig. 4:**
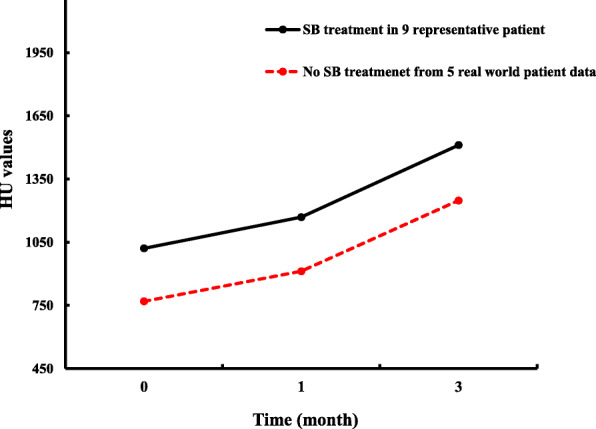
Bone mineral density (BMD) measurements during a standard dental implantation treatment for 9 patients with SB cell treatment compared to a reference set from 5 real-world patient data for the same treatment without SB cells. BMD measurements were taken at the start of dental implantation treatment, and after 1 month and 3 months on follow-up visits. For both data sets, a rapid increase in BMD was observed within the first month compared to the subsequent 2-month period. Data was provided by Dr Chiang, TMUH. Abbreviation: HU – Hounsfield units

Despite the success of the dental implant procedures, some study limitations make it difficult to gauge if an optimal dose of SB cells was given. There was no control group of patients without SB cells. The lack of any dose-dependent effect could be due to saturated levels of SB cells or an insufficient dose to produce any real effect. Nonetheless, the accelerated bone regeneration observed from week 4 leaves open the possibility that SB cells could ameliorate osseointegration. Good bone quality and quantity were observed for a continuous 3 months after dental implantation. One patient from the high dose group of this study (Case 7) had an increase in BMD starting on week 2. BMD continued to accelerate between week 2 and week 8 then gradually flattened after week 12. A common observation for a typical dental implant treatment course is the increase of BMD 1 week after dental implantation which is followed by a plateau effect. From week 16 to week 24, patients from the high dose group had an apparent faster acceleration of BMD measurements when compared to patients in the lower dose groups. As this study had limited patient enrollment, the observed trends between the group data were not statistically significant. In standard treatments conducted at the study site, a rapid increase in BMD is typically observed within the first months after implantation before leveling off at the end of month 3 in Fig. 4. (Discussion with Dr. Chiang of TMUH). The initial rapid increase in bone density also corresponds to what was observed in patients receiving dental implants in the posterior mandible and with no prior guided bone regeneration procedure [16]. That study suggested that SB cells may improve osseointegration with one patient that received SB cells having a faster increase in BMD than those that received standard treatment. The results support previous observations of SB cells to promote bone repair and formation in animal studies.

The mechanism of how SB cells could accelerate bone density also remains unclear. The possible paracrine signaling by SB stem cells would be consistent with the ability of some other stem cells to secrete paracrine factors [30]. From this study, we examined the profiles of cytokines and chemokines during the study period, from pre-treatment of SB cells up to 12 weeks after SB treatment. Dental implantation itself is known to trigger cytokine and chemokine production that are involved in inflammation and bone remodeling. The changes in cytokine and chemokine levels will create unique microenvironments and affect various signaling pathways. These effects may not be captured in the circulating blood or at the selected time points of this study. The elevated levels of inflammatory cytokines over a sustained period could also be partially attributed to the transplantation of SB cells and not due to dental implantation alone. Furthermore, the treatment procedures and heterogeneous patient populations could also influence the measurements and consistence of systematic cytokines and chemokines. Similarly, the variations of cytokines and chemokines test are also observed in other phase I stem cell clinical trials [21, 22]. Thus, the interpretation of potential biological and therapeutic signals of SB cells should be cautious.

The results of cytokines and chemokines demonstrated SB cells do not exacerbate systematic inflammatory response and confirmed the safety of SB cells in localized application. Although no significant differences of most cytokines and chemokines were detected among the selected time points in all groups, six biomarkers (Fracktalk, IL-17A, FGF2, eotaxin, MDC, and MCP-1) revealed similar tendency during 24 weeks of investigation. The decreasing tendency of proinflammatory cytokines (Fracktalk and IL-17A) supported the immunomodulatory effects of SB cells (Fig. 3a). As shown in Fig. 3b, FGF2 can promote vascularization and accelerate the physiological bone healing, which is especially obvious in middle dose group and high dose group. Moreover, eotaxin, MDC, and MCP-1 chemokines are reported to regulate osteoclastogenesis, recruitment of mesenchymal stem cells, osteoblast differentiation, and bone formation [31–33].

An increase in MCP-1 was not only observed in the low dose group and middle dose group but also exhibited an overall dose-dependent effect among all groups. Only patient 2 had a slightly decrease in MCP-1 expression. Based on this positive and dose-dependent trend, MCP-1 could be further investigated as a surrogate marker of SB cells. MCP-1 (also known as CCL2) is a ligand for CCR2 and Duffy antigen receptor for chemokines [34, 35]. The elevated levels of MCP-1 may be related to the observed bone regeneration in this study. It has been reported to mobilize calcium and facilitate bone growth and wound repair by various sources of stem cells [36–39]. For example, MCP-1 was shown to recruit monocytes as well as memory T cells and dendritic cells. During bone remodeling, the recruitment of monocytes was reported to be based on the temporal and spatial expression of MCP-1 by osteoblasts. MCP-1 was reported to facilitate osteoclast recruitment, differentiation, and the fusion of osteoclasts and osteoclast precursors [40, 41]. These findings have been supported by knockout mouse models [42, 43]. However, our small sample size and lack of an appropriate control group need to be cautious for judgment from our results relating to cytokines and chemokines. Thus, the cytokine analysis in this study is limited by low statistical power and the study design. It may be difficult to detect MCP-1 due to its reported short serum half-life. Furthermore, elevated MCP-1 has been observed in a study of patients with titanium dental implants [44]. Therefore, further studies would be needed to confirm changes in cytokine levels and show if MCP-1 is triggered by SB cells, whether directly or indirectly.

As a possible stem cell therapy, SB cells may confer advantages in tissue engineering [45]. For example, the accelerated HU score improvement suggests that SB cells can increase biocompatibility of tissue engineering scaffolds as well as the osseoinductivity to improve bone healing. One important consideration are the interactions between the elastic properties, layer thickness, and porosity of the dental implant material itself [45, 46]. Improving biocompatibility may be enhanced by the SB cells’ ability in forming a thicker oxide layer and porosity, by which to further promote cell attachment, proliferation, differentiation, and adhesion between cells and bones. The relationships between implant materials, stresses, SB cells, and bone regeneration require further study and optimization. In this study, a commercially available collagen-based scaffolding for SB cells was used [47, 48]. New types of 3D printing technologies or other scaffolds could also improve the application of SB cells in the future [49–51].

The use of SB cells in regenerative medicine is especially appealing because cell expansion may not necessary for the application of SB cells in the clinic. Autologous SB cells can be easily collected from human peripheral blood and bone marrow. Other stem cells such as mesenchymal cells that are isolated from bone marrow require more invasive procedures and sometimes require the use of expensive small molecules [52]. The unique features of SB cells support further studies on their efficacy in dental implant procedures. However, the “3 + 3 design” in this phase I study has limitations such as the following: fixed cohort sizes (either 3 or 6), a small number of patients, and lack of an appropriate control group. In addition, the cortical bone thickness and trabecular bone microarchitecture between maxillary and mandibular alveolar bone was different and may influence the healing capacities. To eliminate the bias of different jaw regions in alveolar bone regeneration capacities, only D3 level bone density of patients were enrolled in this clinical trial. Despite these limitations, positive clinical outcomes were both observed in BMD and immune response. Whether the SB cells contributed these outcomes cannot be determined definitively in this phase I study; a well-designed phase II study is needed to prove the dose-related therapeutically benefits.

## Conclusion

Overall, SB cells can potentially increase bone regeneration and therefore hold therapeutic potential in the field of dental implants. This phase I study demonstrated that SB cells were safe and tolerable to patients with severe bone defects. The results also showed acceleration of dental restoration possibly due to SB cells. SB cells may eventually be studied as an alternative for dental implants or other unmet clinical needs in cell and gene therapies and tissue engineering. Further testing of SB cells in phase II clinical trials is warranted.

## Supplementary Information

**Additional file 1: Figure S1**. Clinical illustration and radiographic features of GBR, the implant placement and restoration procedures. (A) The postextraction socket and the related bone defect. (B) The alveolar socket filled with SB cells and bone substitutes. (C) Wound healing after 8 weeks. (D) The well-regenerated structure of alveolar ridge after 12 weeks of healing. (E) implant placement with healing abutment. (F) Definitive metal-ceramic crown at delivery.

**Additional file 2: Figure S2**. The whole assessment schedule of the clinical trial

**Additional file 3: Figure S3**. CD61-Lin- SB cells are systematic and local safety for cell therapy. (A) There was no detectable tumor formation when transplanting different number CD61-Lin- SB cells into immunodeficient NOD-SCID mice (n = 28) by subcutaneous injection. (B) CD61-Lin- SB cells were infused to NOD-SCID mice (n = 10) via tail vein for the systematic safety analysis. There were no abnormal observations in mice, including pain, hair and body weight. (C) No promotion risks of SB cells were presented after subcutaneously implantation of both A549 (lung adenocarcinoma cells) and SB cells into immunodeficient NOD-SCID mice. SB cells have non-tumorigenicity. Taken together, CD61-Lin- SB cells are a systematic and local safe resource for cell therapy.

**Additional file 4: Figure S4**. CD61-Lin- SB cells promote the bone repair in calvarial defects of mice. (A) After 5 weeks, 3 months, and 5 months, the specimens were evaluated by μCT. The mice implanted with collagen sponges alone did not generate any bone compared with the positive control group after 5 weeks’ implantation. (B) the CD61-Lin- SB cells promoted the partial bone formation at 3 months (n = 5) and complete bone regeneration at 5 months (n = 3). (C, D) Results of BMD and BV/TV were shown specifically increasing of the BMD and BV/TV in SB-3 months and SB-5 months. (E) In the SB cell implanted group, the bone formation was observed within the calvarial defect, similar like the positive group implanted hBMP7-hBMSC cells (Fig. 2E).

**Additional file 5: Figure S5**. CD61-Lin- SB cells combined with bone graft (SB cement) promote the bone repair in sinus of rabbits. (A) Surgical procedures for the rabbit sinus. (B) After 2 weeks of healing, more osteoblasts and mineralized matrix was observed in SB cement group when comparing to only bone graft group. (H&E stain) (C) Changes in the sinus volumes between SB cement and only bone graft groups. (D) Changes in the section of the bone defect after CT reconstruction. (E) 1.86 fold increasing of bone density was demonstrated in SB cement group when comparing to only bone graft group after 2 week of healing.

## Data Availability

The data during the current study are available from the corresponding author on a reasonable request.

## Abbreviations

SB cells: Small blood stem cells
CT: Computed tomography
BMD: Bone mineral density
HU: Hounsfield units
GBR: Guided bone regeneration
ROI: Region of interest
AEs: Adverse events
GTP: Good Tissue Practice
ALT: Alanine aminotransferase
Hb: Hemoglobin
BMP-2: Bone mineralization protein-2
