# Supplementary figures and images for: Small blood stem cells for enhancing early osseointegration formation on dental implants: a human phase I safety study
Source: Stem Cell Res Ther. 2021 Jul 2;12:380. doi: 10.1186/s13287-021-02461-z (PMC8254299; doi:10.1186/s13287-021-02461-z)

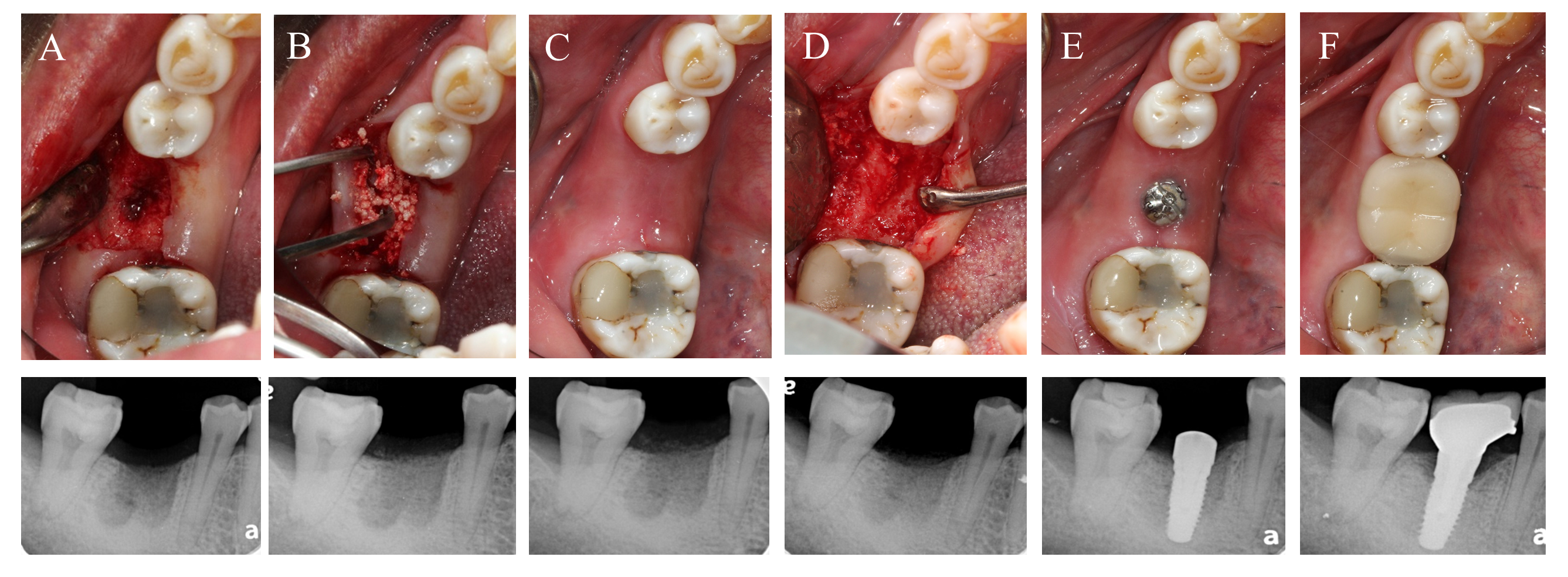

Supplement: Supplementary file 1 — Additional file 1: Figure S1. Clinical illustration and radiographic features of GBR, the implant placement and restoration procedures. (A) The postextraction socket and the related bone defect. (B) The alveolar socket filled with SB cells and bone substitutes. (C) Wound healing after 8 weeks. (D) The well-regenerated structure of alveolar ridge after 12 weeks of healing. (E) implant placement with healing abutment. (F) Definitive metal-ceramic crown at delivery. [file 13287_2021_2461_MOESM1_ESM.tif]

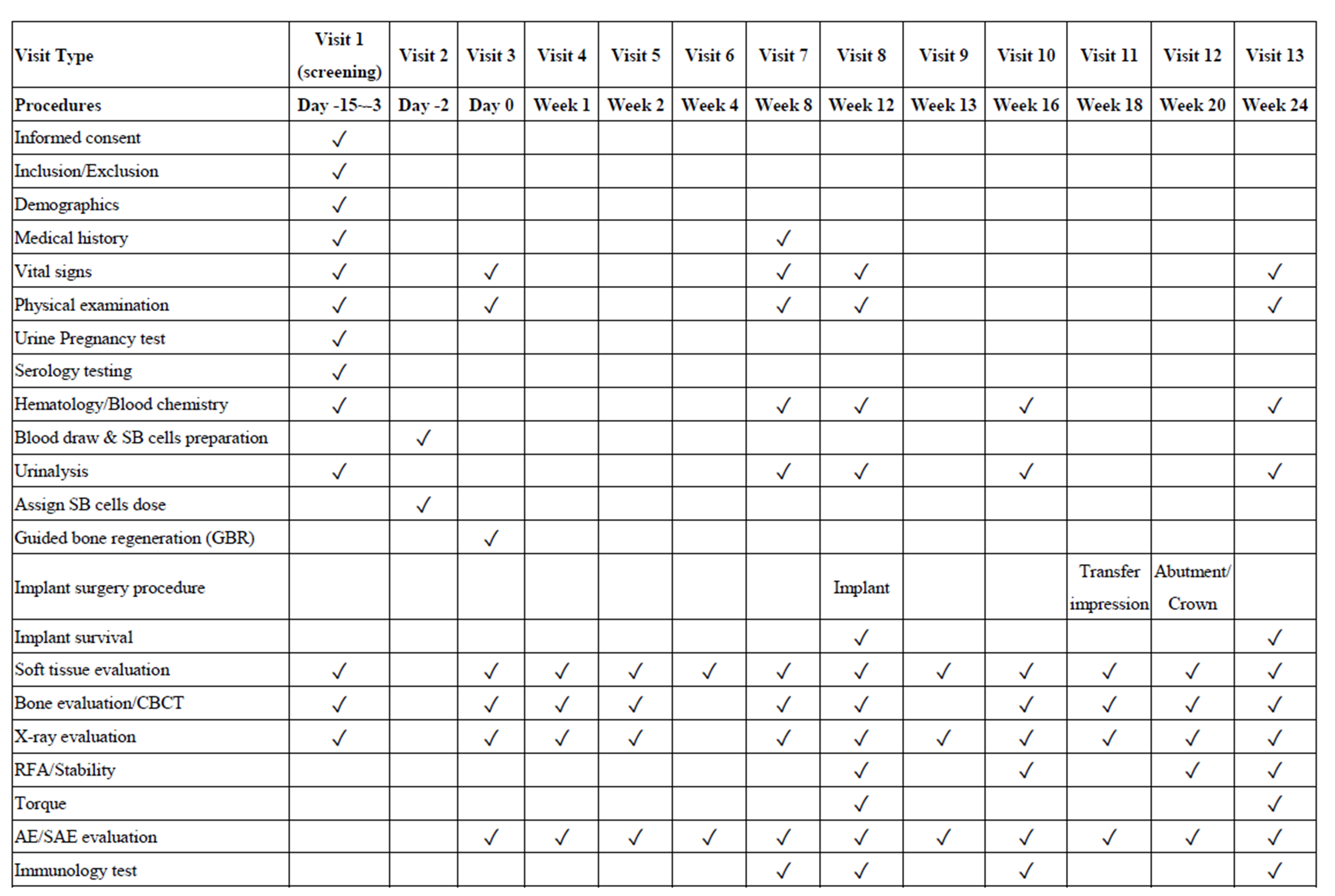

Supplement: Supplementary file 2 — Additional file 2: Figure S2. The whole assessment schedule of the clinical trial [file 13287_2021_2461_MOESM2_ESM.tif]

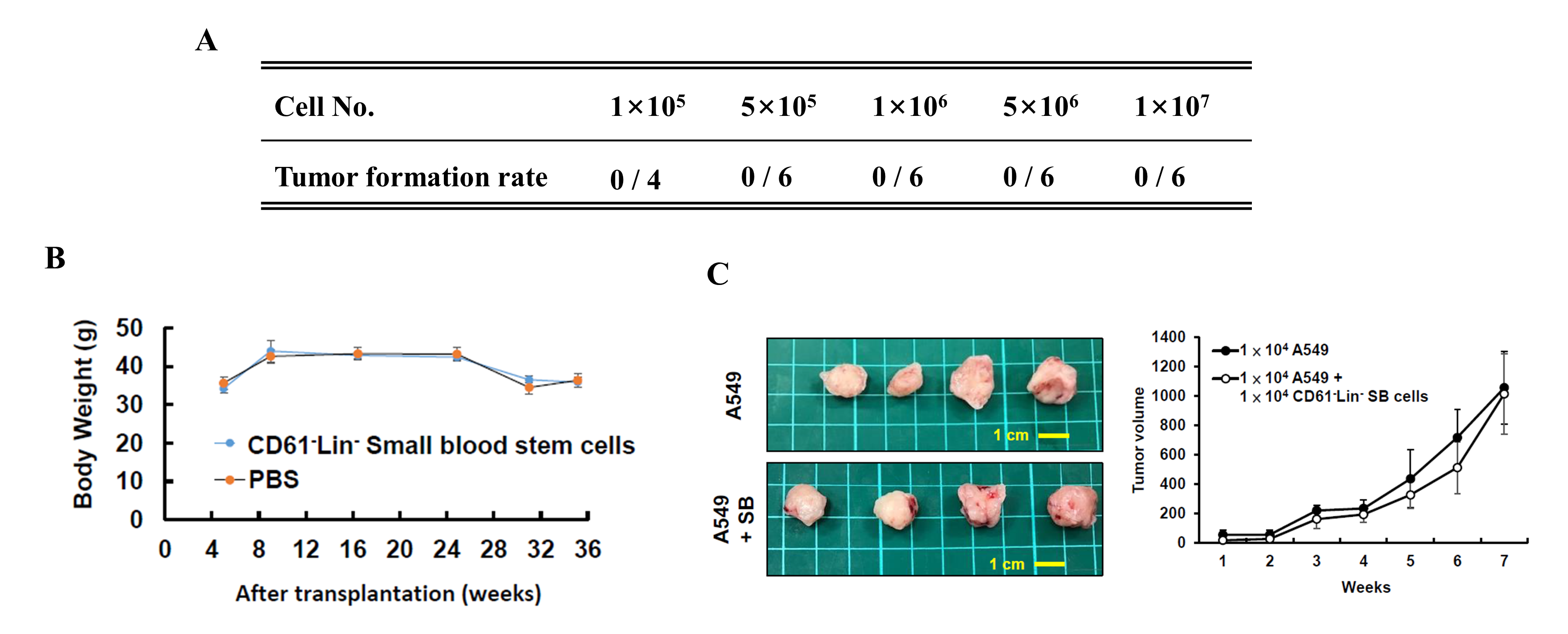

Supplement: Supplementary file 3 — Additional file 3: Figure S3. CD61-Lin- SB cells are systematic and local safety for cell therapy. (A) There was no detectable tumor formation when transplanting different number CD61-Lin- SB cells into immunodeficient NOD-SCID mice (n = 28) by subcutaneous injection. (B) CD61-Lin- SB cells were infused to NOD-SCID mice (n = 10) via tail vein for the systematic safety analysis. There were no abnormal observations in mice, including pain, hair and body weight. (C) No promotion risks of SB cells were presented after subcutaneously implantation of both A549 (lung adenocarcinoma cells) and SB cells into immunodeficient NOD-SCID mice. SB cells have non-tumorigenicity. Taken together, CD61-Lin- SB cells are a systematic and local safe resource for cell therapy. [file 13287_2021_2461_MOESM3_ESM.tif]

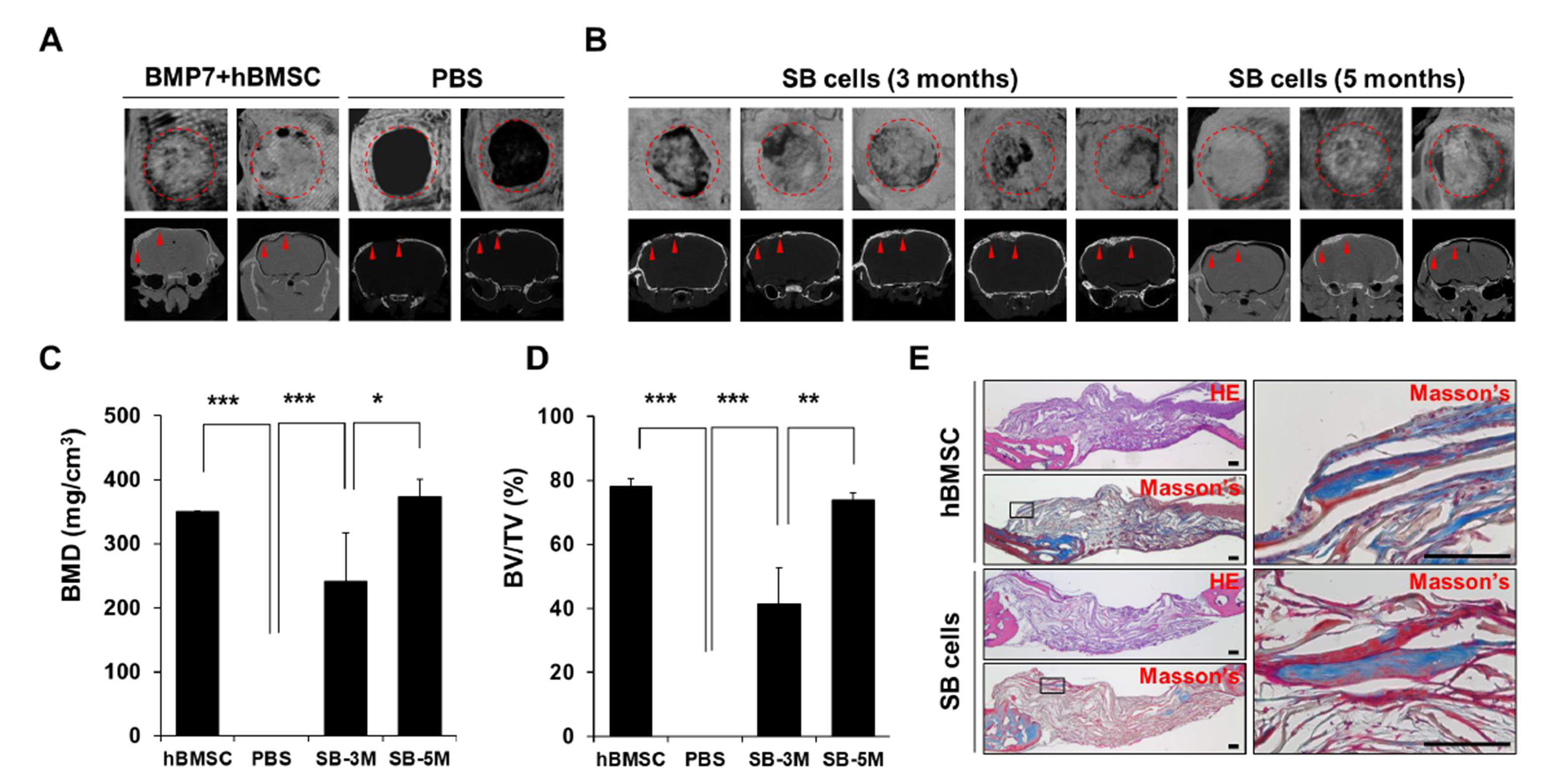

Supplement: Supplementary file 4 — Additional file 4: Figure S4. CD61-Lin- SB cells promote the bone repair in calvarial defects of mice. (A) After 5 weeks, 3 months, and 5 months, the specimens were evaluated by μCT. The mice implanted with collagen sponges alone did not generate any bone compared with the positive control group after 5 weeks’ implantation. (B) the CD61-Lin- SB cells promoted the partial bone formation at 3 months (n = 5) and complete bone regeneration at 5 months (n = 3). (C, D) Results of BMD and BV/TV were shown specifically increasing of the BMD and BV/TV in SB-3 months and SB-5 months. (E) In the SB cell implanted group, the bone formation was observed within the calvarial defect, similar like the positive group implanted hBMP7-hBMSC cells (Fig. 2E). [file 13287_2021_2461_MOESM4_ESM.tif]

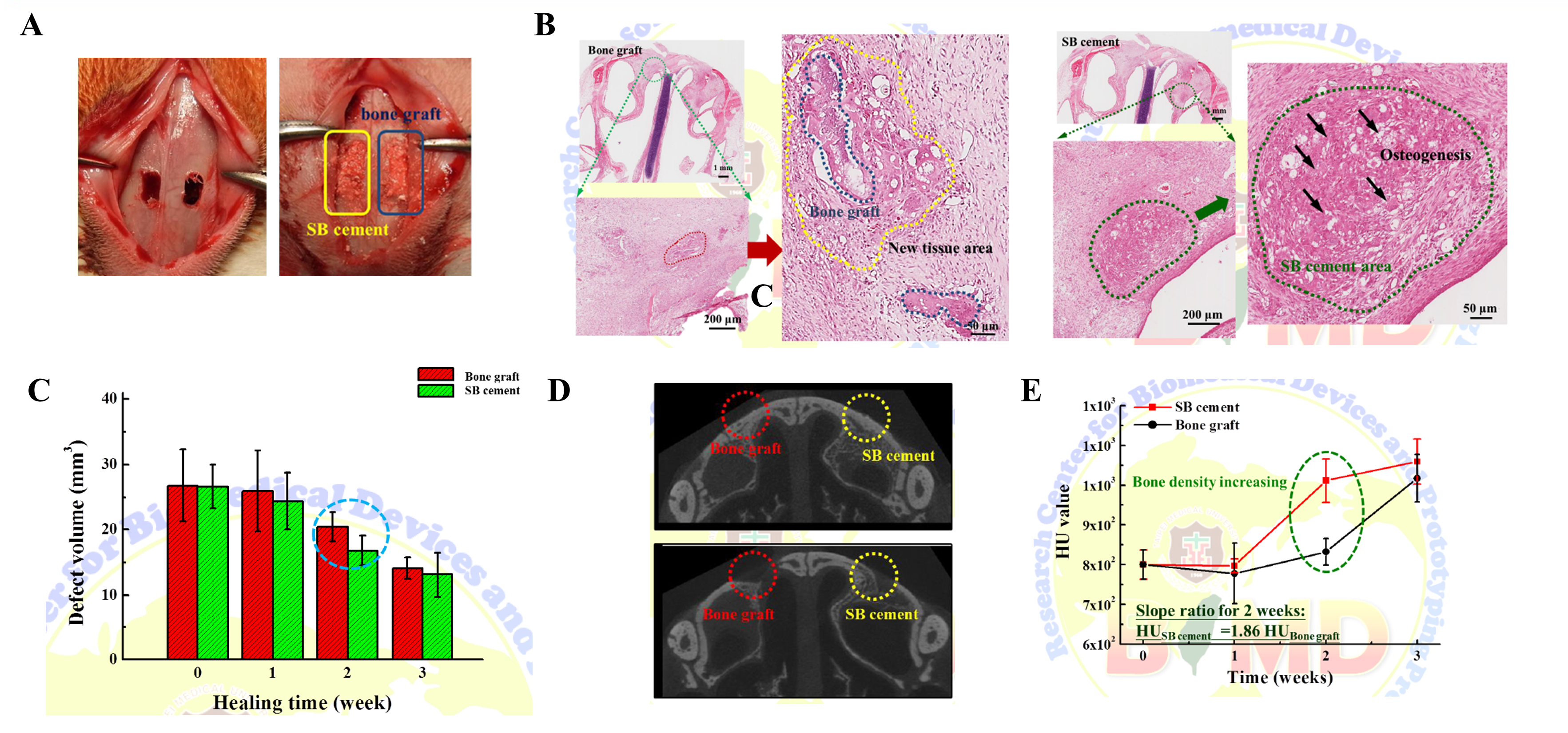

Supplement: Supplementary file 5 — Additional file 5: Figure S5. CD61-Lin- SB cells combined with bone graft (SB cement) promote the bone repair in sinus of rabbits. (A) Surgical procedures for the rabbit sinus. (B) After 2 weeks of healing, more osteoblasts and mineralized matrix was observed in SB cement group when comparing to only bone graft group. (H&E stain) (C) Changes in the sinus volumes between SB cement and only bone graft groups. (D) Changes in the section of the bone defect after CT reconstruction. (E) 1.86 fold increasing of bone density was demonstrated in SB cement group when comparing to only bone graft group after 2 week of healing. [file 13287_2021_2461_MOESM5_ESM.tif]
